# A Diagnostic Challenge in Regional Australia: Concurrent *Mycobacterium avium* Complex Lymphadenitis and Hairy Cell Leukemia

**DOI:** 10.3390/jcm15135174

**Published:** 2026-07-02

**Authors:** Magnus Hanbin Liew, Branavan Sivagnanam, Andrea Chui Rong Chieng, Mohammad Ashraful Islam, Chih-Chiang Hu, Surender Juneja

**Affiliations:** 1 Department of Internal Medicine, Goulburn Valley Health, Shepparton, VIC 3630, Australia; branavan.kandavanamsivagnanam@gvhealth.org.au (B.S.); andreachuirong.chieng@gvhealth.org.au (A.C.R.C.); mohammadashraful.islam@gvhealth.org.au (M.A.I.); 2 Department of Hematology, Peter MacCallum Cancer Centre, Melbourne, VIC 2000, Australia; jason.hu@petermac.org (C.-C.H.); surender.juneja@petermac.org (S.J.)

**Keywords:** hairy cell leukemia, non-tuberculous mycobacteria, *Mycobacterium avium* complex, leukocytoclastic vasculitis, necrotizing lymphadenitis, Kikuchi–Fujimoto disease

## Abstract

**Introduction:** Hairy cell leukemia is a rare, indolent chronic B cell lymphoproliferative disorder characterized by cytopenia, splenomegaly and profound immune dysfunction, predisposing affected individuals to opportunistic infections including non-tuberculous mycobacteria. Concurrent presentation with disseminated mycobacterial infection is un-common and may pose significant diagnostic challenges. **Case Presentation**: We report the case of a 68-year-old Caucasian man with a history of splenectomy who presented with fever, lymphadenopathy, leukopenia and a generalized rash. Initial investigations including infectious, hematological and vasculitis workups were inconclusive. Lymph node histology demonstrated necrotizing lymphadenitis, which in the context of an otherwise negative investigations was initially suggestive of Kikuchi–Fujimoto disease. Subsequently, cultures from lymph node tissue and blood yielded *Mycobacterium avium* complex, establishing a diagnosis of disseminated infection. Further bone marrow evaluation with flow cytometry ultimately confirmed underlying hairy cell leukemia. **Conclusions**: This case highlights how an impaired immune milieu may obscure classic clinical and histopathological features, contributing to diagnostic delay and potentially inappropriate immunosuppressive treatment. Clinicians should maintain a high index of suspicion for underlying hematological malignancy in patients presenting with unexplained cytopenia in association with atypical infections. Early consideration of bone marrow evaluation can be crucial.

## 1. Introduction

Hairy cell leukemia (HCL) is a rare, indolent chronic B cell lymphoproliferative disorder, accounting for approximately 2% of all leukemias [1]. The disease is classically characterized by the clonal proliferation of mature B lymphocytes displaying distinctive, fine cytoplasmic hair-like surface projections [1]. Although early-stage HCL is frequently asymptomatic, progressive neoplastic infiltration of bone marrow and structural splenic sequestration typically results in splenomegaly, profound cytopenia and monocytopenia [2,3]. The clinical sequelae of this hematological failure manifest as debilitating fatigue secondary to anemia, bleeding diatheses driven by thrombocytopenia and an exceptionally high vulnerability to severe and opportunistic infections, resulting from a combination of leukopenia, monocytopenia and dysfunctional B lymphocytes [2]. Histologically, tumor cells are commonly detected within the bone marrow and spleen, and less commonly within the liver, lymph node and peripheral blood [4]. Diagnosis is confirmed by morphological and immunophenotypic features on bone marrow biopsy.

Non-tuberculous mycobacteria (NTM) is a diverse group of ubiquitous, free-living environmental acid-fast bacilli distinct from the Mycobacterium tuberculosis complex and Mycobacterium leprae, which predominantly exploits immunocompromised hosts such as patients with HCL [5]. While NTM infections typically manifest in children or individuals with advanced immunodeficiency, the overlap of its clinical and pathological features with underlying hematological malignancy presents a unique diagnostic conundrum [5].

We report the case of a 68-year-old Caucasian man from regional Australia who presented with a febrile illness, cervical lymphadenopathy, cytopenia and a generalized rash. This case highlights the significant diagnostic challenges encountered in a regional Victorian base hospital and demonstrates the evolving clinical reasoning and management approach required before the underlying dual pathology was ultimately identified.

## 2. Case Presentation

A 68-year-old man presented to the emergency department with one-week history of generalized body ache, fatigue and joint pain involving his knees, ankles, shoulders and elbows. He denied systemic symptoms such as fever, rash, coryzal symptoms, altered bowel habits and urinary symptoms. Importantly, the patient traveled to Sydney and jungle trekked a week prior to onset of his symptoms. He denied insect bites, cat scratches and animal exposure. His past medical history is significant for traumatic splenectomy 50 years ago and a repaired Type A thoracic aortic dissection 2 years ago. He was not on any prophylactic antibiotics for his splenectomy. There was no family history of autoimmune disease or malignancy. He is a non-smoker and social drinker and presented from home with his wife.

His physical examination revealed tender left supraclavicular lymphadenopathy without hepatosplenomegaly. His vital signs were normal on admission. Initial laboratory findings revealed leukopenia, neutropenia, and elevated inflammatory markers including CRP, ESR and procalcitonin. Chest X-ray was normal. CT neck, chest, abdomen and pelvis showed no septic focus but noted superior mediastinal, left supraclavicular and right upper aortocaval lymphadenopathy (Figure 1).

The patient was started empirically on intravenous tazocin for neutropenic sepsis and admitted under the medical team. After admission, the patient subsequently developed intermittent daily fevers, night sweats and a generalized non-blanching maculopapular rash involving the chest and both upper and lower limbs (Figure 2). He remained hemodynamically stable throughout his hospital stay.

Given the presentation of a middle-aged man with fever, cytopenia, lymphadenopathy, generalized rash and recent bushwalking exposure, a broad list of differential diagnoses was considered. Differentials included atypical infections like rickettsia disease, viral illnesses with a glandular fever-like presentation including Epstein–Barr virus (EBV), cytomegalovirus (CMV), HIV, underlying lymphoproliferative disorders and primary or secondary systemic vasculitis.

Comprehensive investigations were undertaken. Besides the finding of leukopenia, neutropenia and elevated inflammatory markers, routine blood tests showed normal hemoglobin, platelets, electrolytes, renal function, coagulation and creatinine kinase levels. Infectious disease evaluation was negative as serial blood cultures remained sterile, and targeted serological and molecular testing for rickettsia species, EBV, CMV, HIV, Bartonella, flaviviruses, Brucella, malaria, viral hepatitis, cryptococcal antigen and latent tuberculosis were non-reactive. Furthermore, autoimmune panels and vasculitis screen including rheumatoid factors, cyclic citrullinated peptide (CCP) antibodies, antinuclear antibodies (ANAs), extractable nuclear antigens (ENAs), and C3 and C4 proteins were unremarkable. Key hematological evaluations such as a hematinic screen, peripheral blood film, peripheral flow cytometry, lactate dehydrogenase (LDH) and a direct antiglobulin test (DAT) demonstrated no abnormalities. The results of the key investigations are summarized in Table 1.

Punch biopsy of the rash demonstrated leukocytoclastic vasculitis. Histological examination of the superficial dermis showed perivascular mixed inflammatory infiltrate composed of lymphocytes and neutrophils, with prominent leukocytoclasia. There were no features suggestive of leukemia cutis and no organisms were identified on Gram or PAS staining.

Excisional biopsy of the supraclavicular lymph node revealed necrotizing lymphadenitis characterized by discrete foci of necrosis with karyorrhectic debris in a background of lymphocytes, plasma cells and crescentic histiocytes (Figure 3 and Figure 4). In immunohistochemistry, CD3 highlighted numerous T cells with CD8 expression. Plasmacytoid dendritic cells at the edge of necrotic areas were also stained with CD123. Neutrophils were absent in the biopsy. No abnormal lymphoid population was detected on flow cytometry of the lymph node biopsy, and no organisms were identified on special stains like ZN, WF, GMS, PAS and Gram.

Despite empirical broad-spectrum antibiotic therapy, the patient continued to experience persistent daily fevers, night sweats, and a progressive rash. Filgrastim was administered to support neutrophil recovery and reduce the risk of infective complications. In the context of negative extensive infectious and autoimmune and hematological workup, and following the identification of necrotizing lymphadenitis on lymph node biopsy, a provisional diagnosis of Kikuchi–Fujimoto disease was made. High-dose prednisolone was commenced which led to initial improvement in symptoms including fever and rash. The patient was subsequently discharged with outpatient medical follow-up.

During gradual tapering of prednisolone in the outpatient setting, his symptoms recurred with intermittent fevers, night sweats, non-productive cough and a generalized rash. This was accompanied by a rise in inflammatory markers and neutropenia, necessitating readmission. Concurrently, extended mycobacterial cultures from the initial lymph node biopsy grew *Mycobacterium avium* complex (MAC), correcting the provisional diagnosis to non-tuberculous mycobacterial lymphadenitis.

Extensive repeat investigations were performed. Blood cultures grew *Mycobacterium avium* complex and repeat CT imaging of the chest demonstrated new scattered, ill-defined ground-glass opacities with peribronchial thickening involving both lungs. The patient was treated for disseminated *Mycobacterium avium* complex infection with moxifloxacin, azithromycin and ethambutol.

Bone marrow evaluation was undertaken to assess for possible underlying immunosuppression and persistent neutropenia. Bone marrow aspirate showed occasional lymphocytes with pale cytoplasm, absent nucleoli and circumferential fine villous cytoplasmic projections (Figure 5).

Flow cytometry on bone marrow sample showed an abnormal B cell population with increased side scatter properties and expressed CD11c+, 19+ (bright), 20+ (bright), 25+, 103+, 123+, and 200+ (bright). Bone marrow trephine biopsy revealed mildly hypercellular marrow with typical extensive interstitial infiltrate of CD20 and BRAF-positive lymphocytes (Figure 6). Small granulomas composed of histiocytes including giant cells, lymphocytes and fibroblasts without obvious central necrosis were seen (Figure 7). ZN stain and cultures were negative. Next-generation sequencing (NGS) performed on a bone marrow aspirate sample detected typical BRAF (V600E) mutation. Overall bone marrow findings, flow cytometry and morphological features ultimately established a diagnosis of HCL with concurrent disseminated MAC infection. The patient was commenced on vemurafenib in parallel with appropriate antimycobacterial therapy.

The patient responded well to antimycobacterial therapy, with resolution of his constitutional symptoms, including fever, generalized rash and lymphadenopathy. However, he subsequently developed mild cholestatic liver dysfunction, likely secondary to antimycobacterial treatment which remains under close monitoring. Following the initiation of vemurafenib, his leukocyte, neutrophil, and monocyte counts recovered and stabilized. A repeat bone marrow biopsy is planned to assess his response to vemurafenib therapy. The patient continues to remain under close multidisciplinary surveillance in the hematology and infectious disease clinics to monitor treatment efficacy and adverse effects. Overall, he has demonstrated significant clinical and biochemical improvement, suggesting a favorable prognosis. A chronological timeline of the patient’s clinical presentation, diagnostic evaluation and management is presented in Figure 8.

## 3. Discussion

Epidemiologically, HCL predominantly affects middle-aged to older adults, with a median age at diagnosis of approximately 50 years old, a marked male predominance (male-to-female ratio of approximately 4:1) and a higher incidence among White populations [6]. The sociodemographic profile of our patient was in keeping with these recognized epidemiological characteristics.

Although the patient in our case exhibited characteristic hematologic features of HCL, including persistent leukopenia, neutropenia and complete monocytopenia, several factors contributed to the delay in performing a bone marrow evaluation. The clinical presentation of our patient—a middle-aged man presenting with pyrexia, cytopenia, lymphadenopathy, rash and recent woodland exposure—initially posed a diagnostic dilemma. The early clinical course was notably atypical for HCL. Constitutional symptoms such as fever and generalized lymphadenopathy are uncommon at presentation, documented in fewer than 10% of cases in the literature [7]. Conversely, palpable splenomegaly, which serves as a diagnostic hallmark and the solitary physical finding in 80% to 90% of HCL case series, was entirely absent in our patient due to a prior splenectomy [7]. This anatomical absence of a classic disease marker combined with the presence of confounding constitutional symptoms, significantly complicated the initial diagnostic trajectory.

In addition to the atypical clinical presentation, the initial investigative workup further obscured the underlying diagnosis. Early investigations for mycobacterial disease, including sputum and blood studies as well as chest imaging, were unremarkable, while definitive mycobacterial culture results remained pending during the acute stages of assessment. Simultaneously, a high suspicion of a primary hematological malignancy was initially downgraded based on a series of negative diagnostic evaluations. Peripheral blood film examination, lymph node biopsy and flow cytometric analysis of both peripheral blood and lymph node aspirates failed to demonstrate clonal or neoplastic population. Crucially, while characteristic hairy cells are typically identifiable on Wright–Giemsa-stained peripheral blood smears in approximately 90% of HCL cases, generally constituting 20% or less of the total leukocyte count, they were entirely undetectable in this patient’s peripheral circulation [4]. These repeatedly unremarkable early investigations led to a temporary deferral of bone marrow evaluation. In retrospect, bone marrow evaluation remains the gold standard for diagnosing underlying hematological malignancy and a negative peripheral hematological workup, including peripheral blood film, lymph node biopsy and flow cytometric analysis of both peripheral blood and lymph node aspirates should not have precluded timely bone marrow assessment. Monocytopenia is a highly sensitive and characteristic diagnostic hallmark of HCL [2]. Its presence early in the patient’s clinical course should have prompted earlier consideration of rare conditions such as HCL and facilitated earlier bone marrow evaluation.

Paradoxically, standard definitive diagnostic interventions such as the lymph node excision biopsy further confounded the clinical picture. While a unilateral cervical mass that is refractory to empirical antibiotic therapy represents a classic presentation of NTM lymphadenitis, the initial histological assessment lacked the pathognomonic features necessary for an early diagnosis [8]. Typically, NTM lymphadenitis is characterized by caseating or necrotizing granulomas alongside mixed granulomatous and pyogenic inflammation [8]. The granulomas are composed of epithelioid histiocytes, and neutrophils may be present in necrotizing areas. However, our patient’s histopathological evaluation revealed necrotizing lymphadenitis, notably devoid of any granulomatous reaction or neutrophilic infiltrate. There were discrete foci of necrosis with karyorrhectic debris in a background of lymphocytes, plasma cells, crescentic histiocytes and CD123-positive plasmacytoid dendritic cells. This specific histological pattern, combined with the absence of initially identifiable infectious etiologies, redirected the provisional diagnosis toward Kikuchi–Fujimoto disease. In retrospect, the absence of well-formed granulomas likely reflected the patient’s severely impaired cell-mediated immunity, which is a consequence of the underlying HCL, compounded by the indolent nature of mycobacterial infection.

The patient in our case was initially treated with high-dose prednisolone for presumed Kikuchi–Fujimoto disease before the diagnosis of MAC lymphadenitis was established. In hindsight, corticosteroid therapy likely resulted in an initial improvement in symptoms through its anti-inflammatory effects; however, it may have also further suppressed an already compromised immune system due to underlying HCL and prior splenectomy. Consequently, corticosteroid use may have contributed to the progression and dissemination of MAC infection in our patient, leading to symptom recurrence that ultimately prompted further diagnostic investigations and unmasked the true underlying pathology.

The systemic immunosuppression observed in HCL is driven by a combination of neoplastic cell phenotypes and a severely compromised immune microenvironment. Mechanistically, the malignant B cells aberrantly express specific surface adhesion molecules, most notably CD11c. Under normal physiological conditions, CD11c is primarily expressed on monocytes and macrophages, where it plays a key role in the recognition, binding and clearance of complement-coated microorganisms [9,10,11]. However, because the neoplastic B lymphocytes in HCL lack the downstream phagocytic and antimicrobial effector mechanisms of professional myeloid cells, they fail to eradicate engulfed pathogens [12]. This functional deficit and the preferential homing of leukemic cells to the spleen and bone marrow instead of lymph nodes allows the leukemic cells to act as protective, permissive niches where intracellular pathogens, particularly Mycobacterium species, can evade host defenses and persist. Furthermore, HCL induces systemic immune dysfunction through NK-cell impairment, bone marrow infiltration and hypersplenism. Collectively, these processes cripple host immune surveillance, predisposing patients to opportunistic and atypical pathogens, particularly disseminated mycobacterial infections [12,13,14,15,16]. In our case, the patient also had a history of traumatic splenectomy which likely further compromised his immune system and predisposed him to more severe infections.

A systematic review by Tirlangi et al. synthesized nearly five decades of the published literature, examining the intersection of mycobacterial infections and HCL [17]. Their analysis of 48 patients revealed that 71% of these cases manifested as disseminated disease, with high rates of lymph node (60%) and pulmonary (56%) involvement, alongside documented mycobacteremia in more than one-third of the cohort, a clinical pattern that closely mirrors our patient’s presentation. The majority of mycobacterial infections were secondary to NTM (69%), and MAC was present in 30% of the patients. Additionally, 90% of the patients had fever, which further reinforces that constitutional symptoms in patients with HCL are an early sign to consider for underlying infections. Although it is possible for mycobacterial infection to precede the diagnosis of HCL, especially in endemic regions, all the reported cases so far have developed mycobacterial infection after an established diagnosis of HCL. This highlights a critical divergence between the established literature and the case presented here, as both pathologies present and are diagnosed concurrently in our case, highlighting the unique diagnostic complexity of our patient’s clinical trajectory. Cutaneous leukocytoclastic vasculitis, as observed in our patient, represents one of the more common non-specific dermatological manifestations in hairy cell leukemia [18]. Notably, despite therapeutic intervention, overall mortality within this population remained high at 40% [17].

According to the 2026 guidelines published by the American Society of Hematology, first-line treatment for HCL consists of purine analog monotherapy with pentostatin or cladribine, which achieves complete remission rates of 70–90% [19]. However, purine analogs are associated with significant myelosuppression and immunosuppression, complicating their use in patients with active infections or severe cytopenias, which were both present in our patient. Consequently, he was commenced on vemurafenib, a targeted therapy associated with a lower risk of myelotoxicity and immunosuppression. Vemurafenib selectively inhibits the BRAF V600E mutation, which is present in more than 95% of patients with HCL [19]. A prospective American study evaluating vemurafenib in patients with relapsed or refractory HCL demonstrated an overall response rate of 100% and a complete remission rate of 42%, supporting its efficacy in the treatment of patients with HCL [20]. This case highlights the importance of individualizing treatment for patients with HCL, as the optimal therapeutic approach depends on factors such as disease burden, hematological parameters and the presence of active infection.

## 4. Conclusions

This case highlights the diagnostic complexity of concurrent HCL and disseminated NTM infection, where atypical clinical features and initially non-diagnostic investigations significantly delayed definitive diagnosis. It underscores the importance of maintaining a high index of suspicion for underlying hematological malignancy in patients presenting with opportunistic infections, particularly when clinical, radiological, and histopathological findings are incongruent. Early consideration of bone marrow evaluation may be crucial in avoiding diagnostic delay and improving patient outcomes in similar presentations.

## Figures and Tables

**Figure 1:**
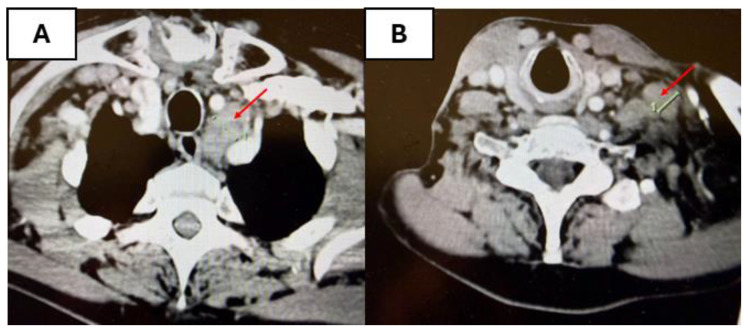
Axial view of CT neck and chest. (**A**) Superior mediastinal lymphadenopathy (red arrow). (**B**) Left supraclavicular lymphadenopathy (red arrow).

**Figure 2:**
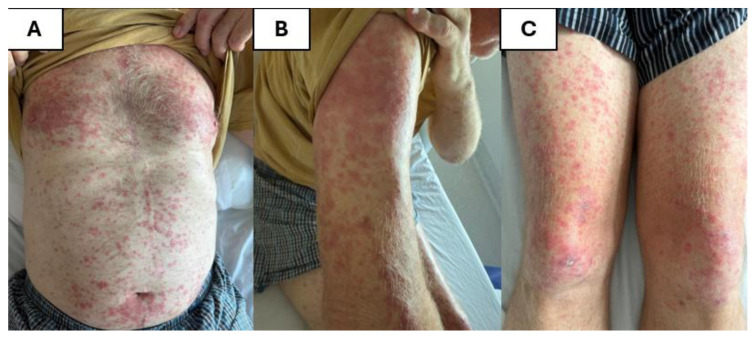
Maculopapular rash. (**A**) Maculopapular rash over chest. (**B**) Maculopapular rash over upper limbs. (**C**) Maculopapular rash over lower limbs.

**Figure 3:**
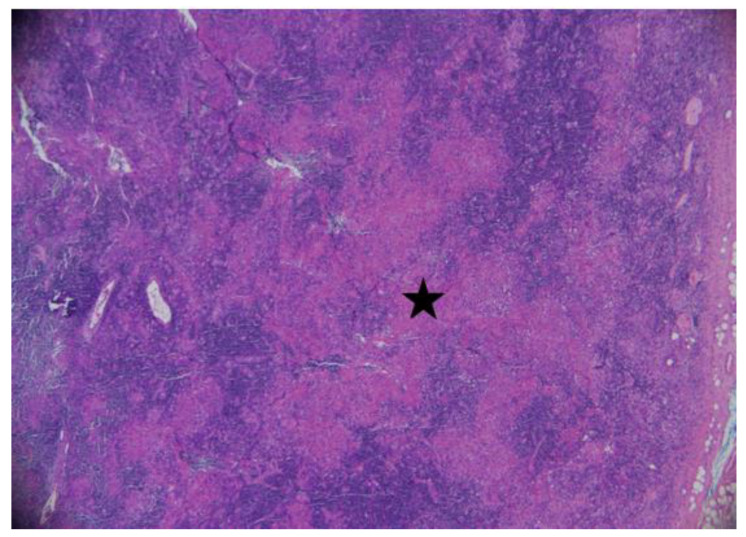
A 4× microscopic view of the supraclavicular lymph node biopsy with Hematoxylin and Eosin staining. The section of lymph node illustrates coalescing areas of necrosis (★) separated by areas of residual lymphoid tissue.

**Figure 4:**
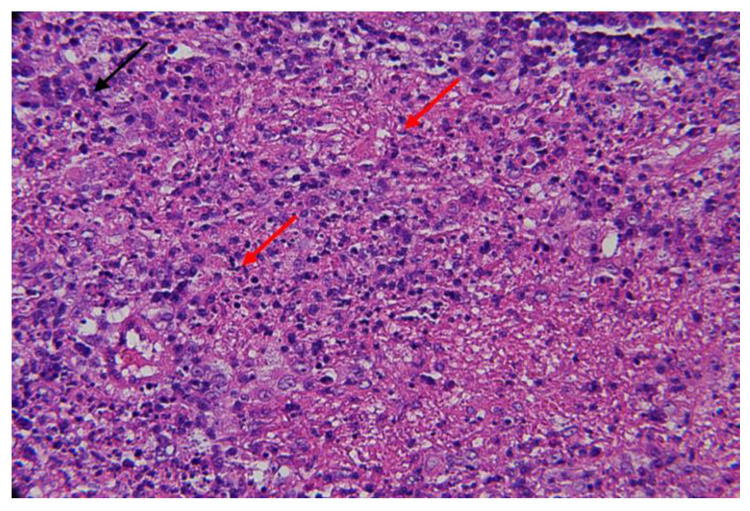
A 40× microscopic view of the supraclavicular lymph node biopsy with Hematoxylin and Eosin staining. The section of lymph node illustrates plasmacytoid dendritic cells, lymphocytes, necrotic debris and crescentic histiocytes (red arrows).

**Figure 5:**
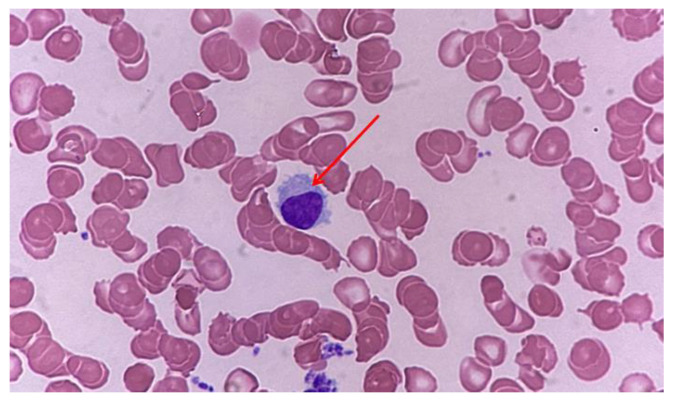
A 40× microscopic view of bone marrow aspirate. Hairy cell with pale cytoplasm, absent nucleoli and circumferential fine villous cytoplasmic projections (red arrow).

**Figure 6:**
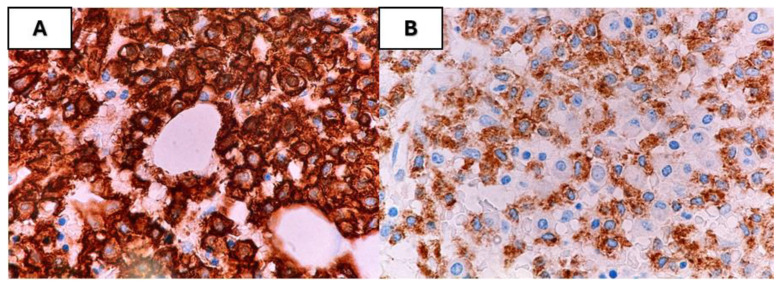
A 40× microscopic view of the bone marrow trephine biopsy sample with IHC and special staining. (**A**) CD 20 staining. (**B**) BRAF staining.

**Figure 7:**
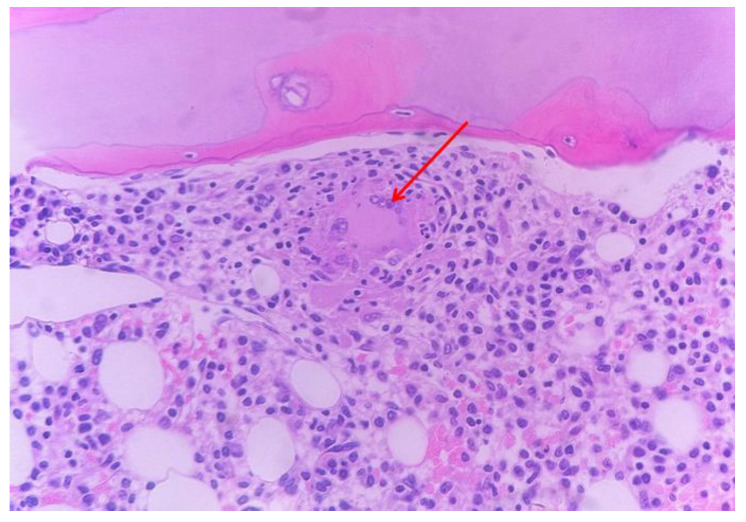
A 20× microscopic view of the bone marrow trephine biopsy. Typical granuloma with histiocytes including giant cells, lymphocytes and fibroblasts without obvious central necrosis (red arrow).

**Figure 8:**
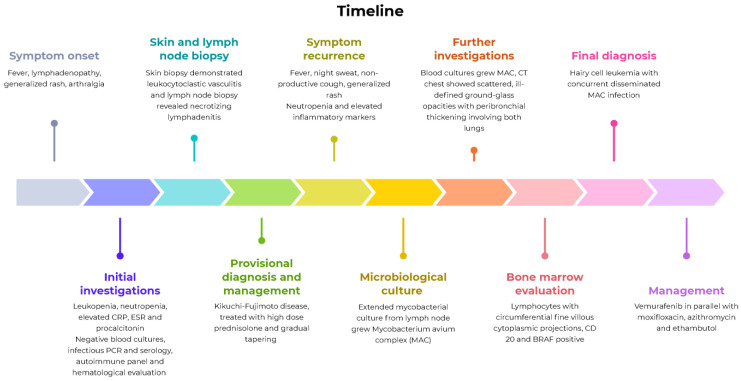
Chronological timeline of the patient’s presentation, diagnostic workup and management.

**Table 1:** Summary of key investigation results.

Blood Test	Result	Reference Range
Hemoglobin	129 g/L	120–170
White cell count	2.0 × 10^9^/L	4.0–12.0
Platelet	219 × 10^9^/L	150–400
Neutrophil	0.8 × 10^9^/L	2.0–8.0
Lymphocyte	1.1 × 10^9^/L	1.0–3.5
Monocyte	0.0 × 10^9^/L	0.2–1.0
Eosinophil	0.0 × 10^9^/L	0.0–0.5
Basophil	0.0 × 10^9^/L	0.0–0.2
Blood film	Moderate neutropenia, asplenic red cell changes noted	
C-reactive protein	82.3 mg/L	<5.0
Erythrocyte sedimentation rate	30 mm/h	1.0–20.0
Procalcitonin	0.39 µg/L	<0.07
Lactate dehydrogenase	231 U/L	120–250
Ferritin	249 µg/L	30–500
Iron	7.8 µmol/L	11.6–31.3
Transferrin	2.11 g/L	1.63–3.44
Transferrin saturation	15%	15–45
Folate	20.2 nmol/L	7.0–46.0
Vitamin B12	193 pmol/L	138–652
Thyroid-stimulating hormone	1.36 mU/L	0.40–4.90
Free thyroxine	14.05 pmol/L	9.00–19.00

## Data Availability

The original contributions presented in this study are included in the article. Further inquiries can be directed to the corresponding author.

## Abbreviations

The following abbreviations are used in this manuscript:

HCL	Hairy cell leukemia
NTM	Non-tuberculous mycobacterium
CRP	C-reactive protein
ESR	Erythrocyte sedimentation rate
CT	Computed tomography
EBV	Epstein–Barr virus
CMV	Cytomegalovirus
HIV	Human immunodeficiency virus
CCP	Cyclic citrullinated peptide
ANA	Antinuclear antibody
ENA	Extractable nuclear antigen
C3	Complement component 3
C4	Complement component 4
LDH	Lactate dehydrogenase
DAT	Direct antiglobulin test
PAS	Periodic acid–Schiff stain
CD	Cluster of differentiation
ZN	Ziehl–Neelsen
WF	Wade–Fite Stain
GMS	Grocott’s methenamine silver
MAC	*Mycobacterium avium* complex
BRAF	B-Raf proto-oncogene
NGS	Next-generation sequencing
IHC	Immunohistochemistry
